# Healthcare utilization in the departments of obstetrics and gynecology during the first two years of the COVID-19 pandemic: time series analysis in Jining, China

**DOI:** 10.1186/s12889-025-22160-1

**Published:** 2025-03-13

**Authors:** Yao He, Hong Xiao, Fang Liu, Xiaochen Dai, Hongyan Wang, Haomin Yang, Zhenhui Liu, Joseph M. Unger

**Affiliations:** 1 Department of Global Health, University of Washington, Seattle, WA USA; 2 Public Health Sciences Division, Fred Hutch Cancer Center, 1100 Fairview Ave N., Seattle, WA USA; 3 Chinese Center for Disease Control and Prevention, Beijing, China; 4 Institute for Health Metrics and Evaluation, University of Washington, Seattle, WA USA; 5 Jining First People’s Hospital, 6 Jiankang Rd, Jining, 272111 China; 6 Department of Epidemiology and Health Statistics, School of Public Health, Fujian Medical University, 1 Xuefu North Rd, Fuzhou, 350122 China; 7 Department of Medical Epidemiology and Biostatistics, Karolinska Institute, 12A Nobels vag, Solna, Stockholm, 17177 Sweden; 8 Affiliated Hospital of Jining Medical University, 89 Guhuai Road, Jining, China

**Keywords:** COVID-19, Health equity, Health services, Obstetrics, Gynecology

## Abstract

**Introduction:**

Healthcare utilization in China decreased precipitously during the initial outbreak of the COVID-19 pandemic, and women were disproportionately affected. As the COVID-19 pandemic has proven to be far more pervasive and persistent than many first surmised, a vital question is whether the utilization of non-COVID related healthcare has remained low under China’s dynamic zero-COVID policy. This study aimed to estimate the initial and enduring collateral effects of the COVID-19 pandemic on the utilization of obstetrics and gynecology care at a tertiary hospital in Jining, Shandong Province, China.

**Methods:**

An interrupted time series analysis was conducted to estimate the impact of the COVID-19 pandemic and mobility restrictions on monthly counts of outpatient visits, inpatient admissions, and surgeries in the obstetrics and gynecology departments at a tertiary hospital in Jining, China. Outpatient visits and surgery volume were abstracted from the hospital’s monthly healthcare delivery report, while inpatient admissions were obtained from de-identified individual electronic medical records of inpatients admitted between January 1, 2017 to December 31, 2021. Incidence Rate Ratios (IRRs) representing monthly service counts compared with counterfactual counts (had the pandemic not happened) and the volume (number) of patients lost due to the pandemic were estimated.

**Results:**

During the study period, there were a total of 1 181 120 outpatient visits, 89 550 inpatient admissions and 49 056 surgeries in the obstetrics department; and 847 124 outpatient visits, 42 644 inpatient admissions and 39 653 surgeries of these totals occurred in the gynecology department. Compared to the expected estimates had the pandemic not occurred, a 55.4% (95% CI: 52.6-57.9%; *p* < 0.001), 31.1% (95% CI: 27.2 − 34.7%; *p* < 0.001), and 27.6% (95% CI: 23.2- 31.8%; *p* < 0.001) decrease was observed in obstetric outpatient visits, inpatient admissions, and surgeries, respectively in the month of February 2020 when the lockdown was enforced; and a 87.4% (95% CI: 86.0 − 88.4%; *p* < 0.001), 74.6% (95% CI: 71.0 -79.2%; *p* < 0.001), and 75.5% (95% CI: 70.9 − 77.8%; *p* < 0.001) decrease was observed in gynecologic outpatient visits, inpatient admissions, and surgeries, respectively. As of December 2021, outpatient (IRR = 0.86; 95% CI: 0.80–0.94; *p* < 0.001), surgery (IRR = 0.88; 95% CI: 0.82–0.95; *p* < 0.001), and inpatient (IRR = 0.73; 95% CI: 0.68–0.79; *p* < 0.0001) services in the obstetrics department, and outpatient visits (IRR = 0.90; 95% CI: 0.82–0.89; *p* = 0.007) in the gynecology department had not fully recovered to pre-pandemic levels. Rural residents experienced a larger immediate decrease in inpatient care utilization in both obstetrics and gynecology in the month of February 2020, and the return to pre-pandemic levels in care utilization was also slower than that of urban residents.

**Conclusions:**

The COVID-19 pandemic led to sizable disruptions in routine delivery and utilization of obstetrics and gynecology care. Disruptions were particularly substantial during the initial wave of the outbreak, and full recovery to pre-pandemic levels has not yet been achieved. The impact was more dramatic for women from rural areas, highlighting the need for policies and programs that address inequities in pandemic response and preparedness.

## Introduction

The World Health Organization (WHO) declared in March 2020 the novel coronavirus (COVID-19) outbreak a pandemic [1]. Disruptions to health systems and routine healthcare services imposed by the COVID-19 pandemic have been repeatedly and widely reported, both in China and worldwide [2–6]. Several reasons have been cited for declining healthcare utilization during the COVID-19 pandemic, including patients’ fear of getting infected in health facilities, suspension or cancellation of non-COVID-19 care, mitigation measures including lockdowns and mobility restrictions, inability to pay for healthcare due to loss of income or health insurance, and increased family responsibility caring for children during the pandemic [3, 6–10].

Owing to stringent non-pharmaceutical interventions (NPIs) and a centralized epidemic response system [1, 11–13], the initial wave of the pandemic in China was quickly controlled. Although subsequent locally transmitted infections in China occurred after containment measures were relaxed, these domestic COVID-19 outbreaks were minor [13]. In response to those subsequent locally transmitted infections, China implemented a “Dynamic zero-COVID” policy until the end of 2022, mandating that cases of infection be kept at or near zero using a strict trace and manage approach [14]. Although strict lockdown measures were effective in suppressing further viral transmission, they exhibited notable collateral effects on daily life and the essential functioning of the health system. Moreover, excessive epidemic control measures imposed by local governments, including extended home confinement time and strict quarantine measures for travelers, set barriers to healthcare access even in regions with few or no confirmed COVID-19 cases [3, 15–17].

Existing evidence indicates that healthcare utilization in China decreased precipitously during the initial outbreak of the COVID-19 pandemic and that the indirect effects of COVID-19 disproportionately affected females [2, 3]. Pregnant women who needed to seek obstetrics and gynecology (OB/GYN) care had suppressed immunity, which would make them more susceptible to developing COVID-19 infections that could affect the fetus and increase the risk of miscarriage, premature birth, and pre-eclampsia [18]. Hospital staff had to provide more supervision and additional testing to monitor both OB/GYN indicators and COVID-19 status [18]. Scheduling physical exams for OB/GYN care posed challenges, as providers had to balance COVID-19 control measures, such as spacing out appointments, with the need to ensure timely exam results.

Considering the prolonged nature of the COVID-19 pandemic, it is critical to understand the magnitude of its impact on OB/GYN care and whether women’s access to essential healthcare services has returned to pre-pandemic levels or remains suppressed. This study aimed to estimate the initial and enduring collateral effects of the COVID-19 pandemic on the utilization of OB/GYN care. Specifically, we aimed to answer the following questions: (1) Did the COVID-19 pandemic lead to reductions in OB/GYN care volume at a tertiary hospital? (2) What was the magnitude of lost OB/GYN care due to the COVID-19 pandemic two years after the outbreak of the pandemic? (3) What was the magnitude of recovery of the OB/GYN care volume?

## Methodology

### Study design and data sources

We conducted an interrupted time series (ITS) analysis [19] using monthly time-series data from 1 January 2017 to 31 December 2021 abstracted from the information system at the Affiliated Hospital of Jining Medical University, a tertiary hospital that provides general and specialized healthcare services to an estimated population of 8.4 million in Jining, Shandong Province of China. Data from 1 January 2017 through 31 January 2020 represented a 37-month pre-pandemic period used to establish baseline time trends in healthcare utilization. The primary outcomes were OB/GYN care volume, namely outpatient visits, inpatient admissions, and surgeries in the two departments, respectively. We abstracted monthly counts of outpatient visits and surgeries from the hospital’s monthly healthcare delivery reports. Monthly inpatient admissions by age group, residence (urban or rural), and diagnosis at discharge and risk were abstracted from de-identified electronic medical records.

### Statistical analysis

Our *a priori* analysis plan included analyses of the impact of the COVID-19 pandemic on the primary outcomes in each department; expected counterfactual forecasts to estimate the lost OB/GYN care volume due to the pandemic; and subgroup analyses of the effect on the primary outcomes by predefined patient characteristics.

We used negative binomial models to examine time trends and their potential changes associated with the onset of the COVID-19 pandemic. We decomposed the overall effect by examining both instantaneous level changes in time (before vs. after a given period) to reflect discontinuity interruptions changes associated with the implementation of lockdown measures in the initial wave of the pandemic, and secondly, the removal of the lockdown measures at the end of the initial wave; and also, slope change associated with the recovery period after the initial wave. As our initial explorations of the form of the long-term trend suggested the appropriateness of the linear assumption, we included a linear effect of time to capture the long-term secular trend. Fixed effect monthly indicator variables were included in all models to account for seasonal effects. An AR(1) structure was used to account for autocorrelation in residual errors [20, 21]. We performed augmented Dickey-Fuller tests on the data of the outcome variables and found that they were not stationary; we used partial autocorrelation function to determine that the lag order was one [20]. The full model equation is below.

$$\displaylines{ E(ln\left( {{Y_t}} \right)) = {\alpha _0} + \left( {\sum\limits_{m = 2}^{12} {{\lambda _m}Month} } \right) + {\beta _1}Tim{e_t} \cr + {\beta _2}COVID1 + {\beta _3}COVID{2_t} + {\beta _4}Pos{t_t} \cr} $$

where $\:{Y}_{t}$ represents each of the three primary outcomes in each clinical department at month $\:t$ (running from 0 to 60); $\:{\alpha\:}_{0}$ represents the model intercept; $\:Month$ is an individual dummy variable indexing month of the year using the month of January as the reference; $\:Time$ represents the months elapsed since the start of the study, January 2017; $\:COVID1$ is the dummy variable indicating February 2020 (i.e., the lockdown period, coded as 1 for February 2020 and 0 otherwise); $\:COVID2$ is the dummy variable indicating months starting from March 2020 when stringent mobility policies were lifted (1 for March 2020 and months after, and 0 otherwise); and $\:Post$ represents the months elapsed since March 2020.

Overall lost healthcare utilization over the two-year pandemic period was computed as the difference between the sum of a given fitted outcome in the presence of the COVID-10 pandemic (i.e., factual estimate) and that in the absence of the pandemic (i.e., counterfactual estimate). We simulated 10,000 predictions per month under each scenario (factual and counterfactual) using the coefficients and covariance matrix of multivariate normal distribution derived from the model [22]. To compute the 95% confidence intervals around these differences, we used the range from the 2.5 and 97.5 percentiles of simulated values. *p*-Values were calculated as the smaller of the proportion of simulated values falling either above or below zero. This value was then multiplied by 2 to represent a 2-sided *p*-value.

We examined whether the pandemic differentially impacted population groups using interaction tests. For the subgroup analyses by age, gynecology patients were categorized into four age groups (under 18, 18–34, 35–54, 55 and above); obstetric inpatients were categorized into three age categories (i.e., under 18, 18–34, 35 and above) [23]. We also conducted the analysis by age-adjusted risk groups, categorizing obstetric patients into high-risk group if they had at least one risk factor other than age (see Supplement). All analyses were conducted using R 4.1.0 and a 2-sided alpha value of 0.05. We followed STROBE reporting guideline for retrospective observational cohort study.

## Results

In the obstetrics department, there were 1 181 120 outpatient visits (34.4% since the pandemic), 89 550 inpatient admissions (30.4% since the pandemic; 0.4% aged < 18 and 18.4% >=35; 64.2% high-risk pregnancy; 35.8% from urban districts), and 49 056 surgeries (30.2% since the pandemic) from 1 January 2017 to 31 December 2021 (Table 1). In the gynecology department, there were 847 124 outpatient visits (38.2% since the pandemic), 42 644 inpatient admissions (43.9% since the pandemic; 87.9% aged 18–54; and 25.2% from urban districts), and 39 653 surgeries (43.5% since the pandemic). As of 31 December 2021, all 260 confirmed COVID-19 cases in Jining were diagnosed before 3 March 2020, and no COVID-19 deaths had been reported.

**Table 1:** Characteristics of patients in the obstetrics and gynecology departments in the study

Characteristics	Number of Patients					Chi-square test*
	All patients	Before the pandemic (January 2017-January 2020)		During the pandemic (February 2020-December 2021)		
		Total (%)^a^	Monthly average	Total (%)^a^	Monthly average	
Obstetrics						
Outpatient	1 181 120	775 102 (88.9)	20 949	406 018 (90.6)	17 653	< 0.001
Surgery	49 056	34 254 (3.9)	926	14 802 (3.3)	644	
Inpatient	89 550	62 360 (7.2)	1 686	27 190 (6.1)	1 183	
Inpatient: Residence						
Rural	54 297	37 596 (64.5)	1 017	16 701 (63.5)	727	0.008
Urban	30 298	20 711 (35.5)	560	9 587 (36.5)	417	
Inpatient: Age (years)						
< 18	396	258 (0.4)	7	138 (0.5)	6	< 0.001
18–34	71 644	50 327 (80.7)	1 361	21 317 (82.5)	927	
>=35	16 169	11 775 (18.9)	319	4 394 (17.0)	192	
Inpatient: Risk group within each age group^b^						
< 18						
Low	124	79 (30.6)	2	45 (32.6)	2	0.69
High	272	179 (69.4)	3	93 (67.4)	4	
18–34						
Low	27 363	19 484 (38.7)	527	7 879 (35.2)	343	< 0.001
High	45 375	30 843 (61.3)	834	14 532 (64.8)	632	
>=35						
Low	4130	3031 (25.7)	82	1099 (25.0)	48	0.5
High	12,039	8744 (74.3)	273	3295 (75.0)	143	
Gynecology						
Outpatient	847 124	523 591 (89.0)	14 152	323 533 (90.0)	14 067	< 0.001
Surgery	39 653	22 390 (3.9)	606	17 263 (4.9)	751	
Inpatient	42 644	23 919 (7.2)	647	18 725 (5.2)	815	
Inpatient: Age (years)						
< 18	572	306 (1.3)	9	266 (1.4)	12	< 0.001
18–34	12 756	7 509 (31.4)	203	5 247 (28.0)	228	
35–54	24 741	13 856 (57.9)	374	10 885 (58.1)	473	
>=55	4 573	2 248 (9.4)	61	2 325 (12.4)	102	
Inpatient: Residence						
Rural	30 973	17 351 (75.9)	469	13 622 (73.5)	593	< 0.001
Urban	10 419	5 504 (24.1)	149	4 915 (26.5)	214	

^a^ Missing and unknown data were not included in calculations of column percentages

^b^ High risk factors include at least one of the following: hypertension, polycystic ovary syndrome, existing diabetes, nephritis, kidney diseases, lupus, hyperthyroidism, obesity, congenital heart disease, hepatitis, tuberculosis, severe anemia, schizophrenia, depression, syphilis, chlamydia, HIV-positive, AIDS, twin fetuses, multiple fetuses, gestational diabetes, pre-eclampsia or eclampsia, placenta previa, placental abruption, preterm, ectopic pregnancy, macrosomia, polyhydramnios, oligohydramnios, premature rupture of membrane, abnormal fetal heart rate

* Chi-square test for the distribution

### Model estimated change in obstetrics

Compared to the counterfactual had the pandemic not occurred, a decrease of 55.4% (incidence rate ratio [IRR] = 0.45; 95% CI: 0.42–0.47; *p* < 0.001) in obstetric outpatient visits, 31.1% (IRR = 0.69; 95% CI: 0.65–0.73; *p* < 0.001) in obstetric inpatient admissions, and 27.6% (IRR = 0.72; 95% CI: 0.68–0.77; *p* < 0.001) in obstetric surgeries was observed in February 2020.

**Fig. 1:**
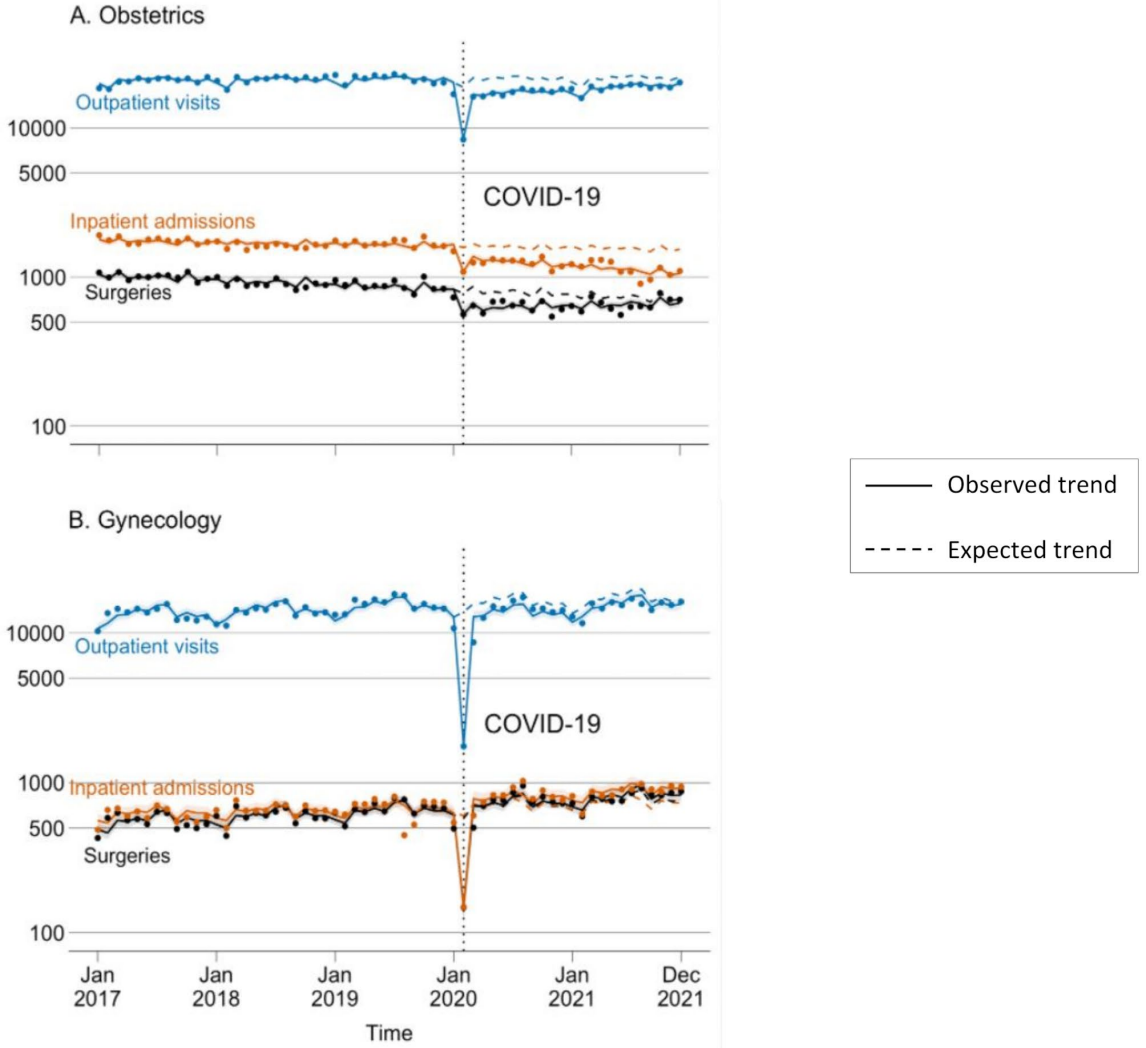
Obstetrics and gynecology outpatient visits, surgeries, and inpatient admissions from January 2017 to December 2021

In March 2020 all three outcomes started to rebound toward expected levels as compared to February 2020 (Fig. 1A; Table 2), but absolute levels remained significantly lower than expected based on pre-pandemic forecasts (outpatient: IRR = 0.78, 95% CI: 0.73–0.82, *p* < 0.001; surgery: IRR = 0.77, 95% CI: 0.71–0.83, *p* < 0.001; inpatient: IRR = 0.82, 95% CI: 0.77–0.87, *p* < 0.001). Starting in April 2020, the quarterly change rate in outpatient visits and surgeries were on average 2.0% (IRR = 1.02 95% CI: 1.02–1.03; *p* < 0.001) and 3.0% (IRR = 1.03; 95% CI: 1.01–1.05; *p* = 0.001), respectively, higher than that had the pandemic not occurred; whereas the quarterly change rate in inpatient admissions was on average 2.0% lower than the quarterly change rate had the pandemic not occurred (IRR = 0.98; 95% CI: 0.96–0.99; *p* = 0.002). The gap between the factual and counterfactual estimates of all three outcomes persisted as of December 2021 (Fig. 1A). During the two-year pandemic period, there was an overall loss of 68 800 (20.2% of the expected level; 95% CI: 14.7- 25.4%; *p* < 0.001) outpatient visits, 5 886 (23.1% of the expected level; 95% CI: 18.6- 27.3%; *p* < 0.001) inpatient admissions, and 2 322 (18.6% of the expected level; 95% CI: 13.5- 23.5%; *p* < 0.001) surgeries in the obstetric department.

**Table 2:** Estimated relative changes comparing estimated healthcare volume in obstetrics and gynecology to the counterfactual had the pandemic not happened

Time period	Feb 2020 (COVID-19 Lockdown)		Mar 2020 (post-lockdown initial re-opening period)		Apr 2020– Dec 2021 (post-lockdown recovery period quarterly change in volume)		Feb 2020– Dec 2021 (overall volume during the period)		Dec 2021 (last month in the observation period)	
Estimates	IRR (95% CI)	*p*-Value	IRR (95% CI)	*p*-Value	IRR (95% CI)	*p*-Value	IRR (95% CI)	*p*-Value	IRR (95% CI)	*p*-Value
**Obstetrics**										
Outpatient	0.45 (0.42, 0.47)	< 0.001	0.78 (0.73, 0.82)	< 0.001	1.02 (1.02, 1.03)	< 0.001	0.80 (0.75, 0.85)	< 0.001	0.86 (0.80, 0.94)	< 0.001
Surgery	0.72 (0.68, 0.77)	< 0.001	0.77 (0.71, 0.83)	< 0.001	1.03 (1.01, 1.05)	0.001	0.81 (0.77, 0.87)	< 0.001	0.88 (0.82, 0.95)	< 0.001
Inpatient	0.69 (0.65, 0.73)	< 0.001	0.82 (0.77, 0.87)	< 0.001	0.98 (0.96, 0.99)	0.002	0.77 (0.73, 0.81)	< 0.001	0.73 (0.68, 0.79)	< 0.001
**Gynecology**										
Outpatient	0.13 (0.11, 0.14)	< 0.001	0.82 (0.72, 0.94)	0.004	1.02 (0.99, 1.05)	0.164	0.82 (0.74, 0.90)	< 0.001	0.90 (0.82, 0.99)	0.007
Surgery	0.25 (0.22, 0.29)	< 0.001	1.01 (0.89, 1.14)	0.932	1.01 (0.99, 1.04)	0.336	1.00 (0.91, 1.09)	0.458	1.07 (0.96, 1.18)	0.881
Inpatient	0.25 (0.21, 0.29)	< 0.001	1.09 (0.94, 1.26)	0.263	1.02 (1.00, 1.05)	0.107	1.10 (0.95, 1.25)	0.902	1.20 (1.03, 1.39)	0.989

### Model estimated change in gynecology

The gynecologic outpatient visits, inpatient admissions, and surgeries in February 2020 decreased by 87.4% (IRR = 0.13; 95% CI: 0.11–0.14; *p* < 0.001), 75.5% (IRR = 0.25; 95% CI: 0.21–0.29; *p* < 0.001), and 74.6% (IRR = 0.25; 95% CI: 0.22–0.29; *P* < 0.001), respectively (Fig. 1B; Table 2). In March 2020, gynecologic surgeries and inpatient admissions rebounded to expected levels (surgery: IRR = 1.01, 95% CI: 0.89–1.14, *p* = 0.93; inpatient: IRR = 1.09, 95% CI: 0.94–1.26, *p* = 0.26). In contrast, gynecologic outpatient visits were still 18.1% lower (IRR = 0.82; 95% CI: 0.72–0.94; *p* < 0.005) than the expected level had the pandemic not occurred.

Starting from April 2020, the monthly change rates in outpatient visits, inpatient admissions, and surgeries were comparable to those had the pandemic not occurred (Table 2). During the two-year pandemic, the factual estimate of outpatient visits was significantly lower than the counterfactual estimate (212 396 versus 259 653; 81.8% of expected; 95% CI: 74.4%, 89.8%; *p* < 0.001).

The factual estimate was comparable to the expected volume for inpatient admissions (11 307 versus 12 337; 109.5% of expected; 95% CI: 0.95–1.25; *p* = 0.90) and surgeries (11 391 versus 11 328; 99.6% of expected; 95% CI: 0.91–1.10; *p* = 0.46).

### Subgroup analysis

**Fig. 2:**
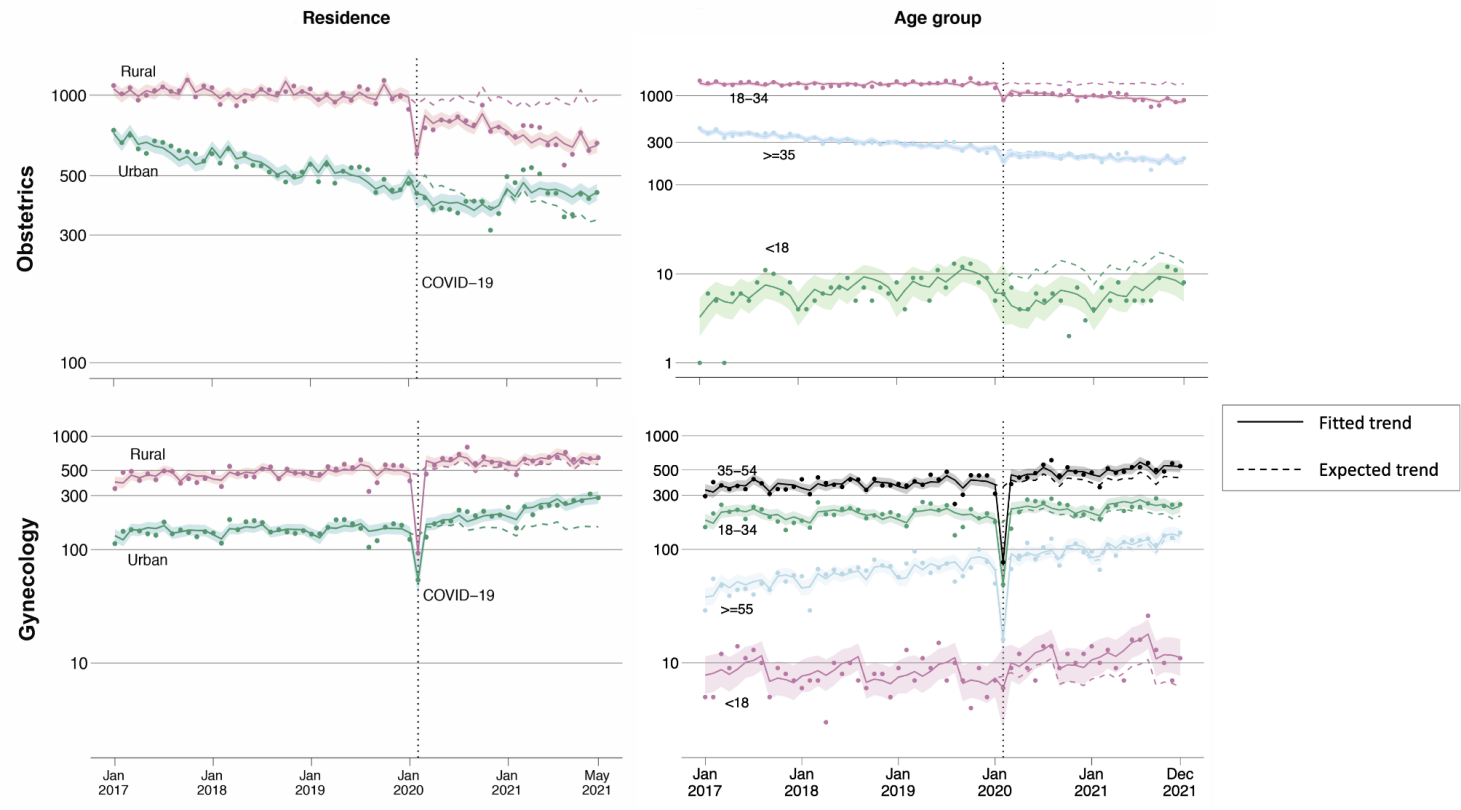
Obstetrics and gynecology inpatient admissions by residence and age group from January 2017 to December 2021

Compared to urban residents, immediate decreases in inpatient care among rural residents were much more profound in both the obstetrics (Ratio of IRR = 0.69; 95% CI: 0.56, 0.84; *p* < 0.001) and gynecology care settings (Ratio of IRR = 0.48, 95% CI: 0.42–0.56, *p* < 0.001) during the lockdown, and the subsequent recovery was slower (Ratio of IRR [obstetrics] = 0.97, 95% CI: 0.96–0.97, *p* < 0.0001; Ratio of IRR [gynecology] = 0.97, 95% CI: 0.97–0.98, *p* < 0.001) (Fig. 2).

Age was also associated with the magnitude of the impact. Immediate decreases in obstetric inpatient admissions among the ≥ 35 age group were smaller (Ratio of IRR = 1.19, 95% CI: 1.09–1.30, *p* < 0.001) during the lockdown compared to those the 18–34 age group. In addition, recovery rate was higher among the ≥ 35 age groups immediately following discontinuation of lockdown measures (Ratio of IRR = 1.13, 95% CI: 1.05–1.22, *p* = 0.004) and during the successive recovery period (Ratio of IRR = 1.02, 95% CI: 1.01–1.02, *p* < 0.001). The patterns that larger decreases during the lockdown and incomplete recovery of obstetric inpatient care as of December 2021 among the 18–34 age group were observed in both low and high-risk pregnant women. However, the effects of the pandemic on inpatient obstetric care for high-risk pregnant women were comparable to those for low-risk women, both in terms of immediate and gradual impacts, across all age groups.

In the gynecologic inpatient department, patients aged 18 and above experienced larger immediate decreases compared to adolescents (Ratio of IRR = 0.36 [ages 18–34 vs. < 18], 95% CI: 0.22–0.59, *p* < 0.001; Ratio of IRR = 0.28 [ages 35–54 vs. < 18], 95% CI: 0.17–0.45, *p* < 0.001; Ratio of IRR = 0.30 [ages ≥ 55 vs. < 18], 95% CI: 0.17–0.54, *p* < 0.001) during the lockdown (Fig. 2).

## Discussion

Our findings showed substantial decreases in outpatient visits, inpatient admissions, and surgeries in the obstetrics and gynecology departments at a tertiary hospital in Jining since the start of the COVID-19 pandemic. Decreases were most noticeable at the outset of the COVID-19 pandemic in February 2020 when a lockdown was in place. All types of services in the two departments started to rebound in March 2020 when the lockdown ended, but only gynecologic inpatient admissions and surgeries recovered to expected levels as of December 2021. The relative reductions in inpatient admissions at both departments were more profound, and the recovery of obstetric inpatient admissions among rural residents slower than that among urban residents.

Our findings are similar to those reported from other countries but differ from those reported by select hospitals in high-income countries. A national survey of obstetricians and gynecologists in China reported reductions in overall clinical activities, especially at tertiary hospitals and general hospitals [24]. Our finding of decreased obstetrics outpatient care are consistent with a systematic review that identified a significant decrease in antenatal visits and unscheduled maternal care visits globally [25]. Another systematic review on maternal and child healthcare services in low- and middle-income countries also found that service utilization decreased compared to the pre-pandemic levels [26]. Similarly, a U.S.-based study on gynecologic and obstetric services at two hospital systems used segmented regression and identified a sharp initial decline followed by gradual recovery, mirroring the trends observed in our study [27]. However, the other two studies conducted in the U.S. and a study in Italy about obstetric services at public hospitals did not find statistically significant changes in service volume associated with the pandemic [28, 29]. Our analyses also revealed a disproportionate impact on healthcare utilization among patients in rural areas, consistent with other studies examining the impact of the pandemic on healthcare utilization in China [2, 3].

During the lockdown implemented in February 2020, utilization of obstetric and gynecologic services decreased significantly at Jining Hospital. Although these departments continued to provide services throughout the pandemic [30], lockdowns and movement restrictions limited patient’s ability to seek care at hospitals, resulting in reduced utilization [3]. Healthcare utilization was impacted not only in obstetric and gynecologic services but also in other departments such as pediatric and cardiology at Jining Hospital [2]. Similar decreases have been observed in other regions of China during periods of COVID-19-related movement restrictions. A cohort study showed considerable reductions in all outpatient and inpatient visits across China in February 2020 when most major cities were locked down [3]. A systematic review of studies from 20 countries including China also found 42% reduction in outpatient visits and 28% reduction in admissions [31].

Following the end of the lockdown in March 2020, service utilization rebounded, but utilization of all obstetric services and gynecologic outpatient services remained below expected rates for over 1.5 years. This could be attributed, in part, to continued non-pharmaceutical interventions against COVID-19 that restrict mobility. These mandatory measures include completion of contact tracing forms before entering hospital; inspection of smartphone Quick Response (QR) codes for travel history at the entrance of all public spaces and transportations; and the requirement for proof of negative results of COVID-19 polymerase chain reaction (PCR) testing [32]. Such measures are likely to disproportionately impact individuals with low technology literacy. Additionally, the potential stress involved in complying with the mandatory measures could also deter some people from seeking care that would otherwise be more accessible [33]. While the recovery in gynecologic outpatient services was slow, gynecologic inpatient admissions and surgeries recovered to pre-pandemic levels more rapidly. This discrepancy might be attributed to perceived urgency around gynecologic surgeries and procedures that required hospitalization [34], and patients in need of these procedures could not go to lower levels of the health system.

The emerging popularity of telehealth consultations may partially explain the incomplete recovery of outpatient visits in the two departments as of December 2021. A cross-sectional survey in China found that OB/GYN issues were the most common reason for online consultations on China’s largest telehealth platform [35]. These OB/GYN issues that prompted online consultations were predominantly mild and included routine antenatal check-ups, menstruation, and vaginitis; patients were typically between 19 and 35 years old (i.e., reproductive age) and tech-savvy [35, 36]. As Jining Hospital almost exclusively provided in-person consultations during the study period, OB/GYN patients with milder health issues in the Jining municipality may have opted for telemedicine platforms elsewhere as the pandemic persisted. The widespread adoption of telemedicine in OB/GYN since the pandemic has also been present in the other countries [37], indicating a potential shift in the norm of OB/GYN outpatient care, particularly during pandemics, natural disasters, and other extreme circumstances [38, 39]. The decline in underlying demand for OB/GYN care, such as a decrease in the fertility intention or birth rate during the pandemic, may also have contributed to the reduced utilization of obstetrics services [40, 41]. According to World Bank data, China’s crude birth rate decreased from 13 per 1,000 people in 2017 to 7 in 2022 [42].

Rural patients were more likely to miss out on obstetric and gynecologic inpatient care during the lockdown. Additionally, rural patients faced a slower resumption of healthcare utilization after the lift of the lockdown. This may be due to rural residents being less technologically literate than their urban residents, which made it harder for them to navigate post-lockdown regulations. Existing systematic inequalities in China have been exacerbated during public health emergencies, making rural populations more vulnerable [43]. A cohort study conducted in seven provinces in China showed that rural households experienced higher rates of unemployment and loss of income due to the COVID-19 pandemic; about 20% of the surveyed households postponed health care seeking following the lockdown in February 2020 [44]. Fear of infection and travel restrictions were main reasons for the delay [44]. However, with reduced income, limited government aid, and inferior health insurance that triggers high out-of-pocket payments for inpatient care in tertiary hospitals, rural residents might be less likely to be able to afford healthcare, particularly high quality care provided at tertiary hospitals, compared to the pre-pandemic period [44, 45]. Our finding prompts critical reflection whether stringent measures that prioritize viral containment may have sacrificed other health outcomes, particularly for vulnerable populations [46]. Policies and referral systems should be in place to strengthen rural healthcare capacity, reduce barriers for rural residents seeking OB/GYN care locally, and ensure timely referrals to tertiary hospitals in urban areas when needed.

The recovery of obstetric services in outpatient, surgical, and inpatient settings has been slower compared to gynecology services due to two possible reasons. First, since the onset of the pandemic, pregnant women have increasingly sought antenatal care online and opted to give birth at secondary hospitals or community hospitals [36]. This has led to a situation where pregnant women in need of hospital care may have been referred from tertiary hospitals to local hospitals for services and follow-up care. This referral may have continued after the lockdown to limit the number of inpatient admissions and maintain physical distancing. The second possible explanation for the slow recovery in obstetric services is the accelerated decline in birth rates in China since the pandemic began. This trend is consistent with the sharp decrease in birth rate observed during the initial stages of the pandemic [47, 48].

During epidemics, balancing pathogen control with the delivery of essential health services, particularly in rural areas, is critical to mitigating broader public health impacts. The WHO operational guidance underscores the importance of adapting healthcare systems by prioritizing maternal and newborn health services to prevent complications and mortality [49]. Recommended measures include regular adjustment of public health interventions based on local epidemiology, maintaining supplies, and leveraging digital platforms to ensure continuity of care. Specific strategies such as consolidating all relevant care in a single in-person visit, supporting self-sampling, and providing remote diagnostic and counseling support, and tailoring treatment plans for chronic conditions [49, 50]. These tailored approaches highlight the need for resilient health systems capable of sustaining equitable access to essential services during health crises.

The strengths of our study lie in the considerably large population and extended period of follow-up, allowing the control for secular trends, seasonal patterns, and potential unobserved time-varying confounding factors. The data used in this study were aggregated from electronic medical records, which are more timely and accurate than other data sources, such as routine public health data or survey data. These records are also more complete and have fewer instances of misclassifications [51]. This study has several limitations. First, the findings were based on in-person healthcare utilization in one tertiary hospital, limiting the external validity of the findings. Thus, although the magnitude of the findings for this large tertiary hospital were notable, and may reflect broader systemwide impacts, the magnitude of the findings likely varies among different types of hospitals throughout China. Second, subgroup analyses by residence, age, or risk group were only conducted among inpatients due to the lack of individual level data for outpatients or patients that had surgeries. A more granular analysis of outpatient and surgical patients could have provided additional insights into specific populations that were most affected by the pandemic and the underlying reasons. Third, while the study examined healthcare utilization, it did not capture potential changes in the quality of care during the pandemic, which could be a crucial factor influencing health outcomes. Additionally, the study lacks longer-term follow-up to assess the sustained effects on outcomes. Lastly, this study did not incorporate patient perspectives, which could provide valuable context to the quantitative findings and highlight specific barriers women faced in accessing obstetric and gynecologic care during the pandemic. Future research incorporating qualitative data from patients could offer deeper insights into these barriers and inform more effective healthcare policies.

## Conclusion

The COVID-19 pandemic significantly disrupted the routine delivery and utilization of obstetrics and gynecology care in a large tertiary care center in China. The disruptions were particularly substantial during the lockdown in the initial wave of the outbreak, with slow recovery attributable to stringent COVID-19 containment measures. Disproportionate impact on rural patients underscores the urgency of implementing of implementing policies to address inequities in pandemic preparedness and response.

## Data Availability

The datasets generated and analyzed and the analysis code that was used during the current study may be obtained from Dr Hong Xiao upon request.
